# Exploring print media coverage of elite athletes’ mental illness between 2010 and 2023 in Germany: a quantitative content analysis

**DOI:** 10.3389/fspor.2024.1446680

**Published:** 2024-10-08

**Authors:** Marcia Hapig, Guido Zurstiege, Jochen Mayer, Ansgar Thiel, Jannika M. John

**Affiliations:** ^1^ Department of Social Sciences of Sport, Institute of Sports Science, University of Tübingen, Tübingen, Germany; ^2^ Department of Empirical Media Research, Institute of Media Studies, University of Tübingen, Tübingen, Germany; ^3^ Department of Sport Sciences, Institute for Health Sciences, University of Education Schwäbisch Gmünd, Schwäbisch Gmünd, Germany

**Keywords:** media coverage, mental illness, elite athletes, responsible journalism, mental health awareness

## Abstract

**Objectives:**

Recently, the stereotype of elite athletes' invulnerabilty has begun to be challenged by an increasing number of elite athletes who talk openly about struggling with mental health. Relatedly, previous research has focused primarily on specific incidents like the media's portrayal of personal disclosures. The purpose of this study was to expand this perspective and give a systematic overview of media coverage related to elite athletes' mental illness by examining more than one decade (2010–2023) of German print media reporting. Specifically, we were interested in changes over time and between broadsheet and tabloid press regarding content-related and formal aspects.

**Methods:**

Based on a systematic search and screening process in eleven German newspapers and magazines, 699 print media articles were analyzed with a codebook, forming a framework of content-related (reported mental disorder; central thematic focus; sources of comments and quotations; perspectives on the high-performance sports system) and formal categories (article genre; elements of responsible journalism). Univariate analyses and binary logistic regression models were used to examine changes over time (2010–2016 vs. 2017–2023) and differences between types of press (tabloid vs. broadsheet press) regarding content-related and formal characteristics.

**Results:**

The results indicate an enhanced awareness towards the topic of mental illness and those affected in recent years within German print media. This was demonstrated by the increased integration of responsible reporting elements, the inclusion of diversified perspectives and the considerate selection of content. Despite this positive trend over time, the findings also suggest that media reporting in the tabloid press bears an increased risk for inappropriate storytelling, focusing primarily on personal tragedies.

**Conclusion:**

As personal fate of prominent figures like elite athletes will always meet great interest in the public, it is of utmost importance that the media report responsibly and promote critical thinking in society. The study shows the media's willingness to question conventional ideals embedded in the sports culture and take a more critical approach to the topic of mental illness in high-performance sports. By demonstrating a greater understanding of the importance and the seriousness of the issue, the media might also contribute to improved mental health awareness in society.

## 1 Introduction

The traditional picture drawn by the media as well as within the sports system presents athletes as self-confident, disciplined, unbreakable and faultless (1, 2). However, high-performance sport constitutes a rather closed setting to which the general public has limited access, often only through media representations. To keep up the idealized image of a mentally strong athlete, those elite athletes affected by mental health problems often stay quiet for fear of stigmatization and loss of credibility (1, 3, 4). The biased but still common association between weakness and mental illness leads to the assumption that being labeled as “mentally ill” (5, p. 150) constitutes the absolute “antithesis of what athletes want to portray” (6, p. 10).

Recently, the prevalent stereotype of elite athletes' invulnerabilty has begun to be challenged by an increasing number of elite athletes who talk openly about struggling with mental health problems on their social media channels or within the traditional mass media (4, 7). A prominent leader of this ongoing process is the Olympian Michael Phelps. While still competing, Phelps shared his personal story about anxiety, depression, and suicidal thoughts with the public, at a time when mental health problems were still silenced in public discourse (8). The French Open as well as the Olympic Games in Tokyo 2021 turned out to reinforce the discussion about mental health risks elite athletes face, with international stars Simone Biles and Naomi Osaka withdrawing from competition to protect their mental health (9, 10). Although the initiation of public disclosures is not always solely in the hands of the athletes themselves, but can be coerced to some extent (e.g., Simon Biles felt compelled to disclose her attention-deficit hyperactivity disorder after a cyberattack on the World Anti-Doping Agency in 2016), such authentic stories can be regarded as emblematic of a gradual shift towards more openness and acceptance for sensitive issues like mental illnesses being discussed openly within society (7–10). As prominent figures in the world of high-performance sports have emphasized the importance of mental health, media portrayals of affected athletes have recently become a focus of media-related research (9, 11–13).

In this context, the public's perception of and reaction to athletes' disclosures appears to be strongly shaped by the way the media connotes their statements (14, 15). Due to the use of familiar narratives that conform to cultural norms and societal expectations, journalists tend to unintentionally present a selective reality of facts, leading audiences to unconsciously adopt prescribed values, ideas and expectations conveyed by media texts (16–18). Thereby, the media exert a strong and systematic impact on its recipients (19) and are attributed with possessing the power to enhance or reduce societal stigma towards sensitive issues like mental illness (20, 21). For instance, Gwarjanski and Parrott (22) found severe mental illnesses like schizophrenia to be frequently associated with violence and criminal behavior in news stories, leading to social alienation from those affected. In contrast, Ross et al. (23) reported an increased willingness to support people living with a mental illness when media content focused on the successful recovery from severe mental illness. It has widely been discussed how mass media content and the public discourse interact and interfere with each other (20, 24). According to a variety of media analyses, public discourse shapes mass media content but is also influenced by media reporting, resulting in an interactively constructed reality (19, 20).

Despite the media's substantial role in influencing public opinion, exploring media coverage of elite athletes' mental illness presents a relatively young research area (14, 25). In light of current developments, studies examining media depictions of elite athletes' disclosures of mental health problems have increased considerably (4, 7, 26). However, most studies focused on single incidents, rather than providing an analysis of the overall media coverage related to mental illness in high-performance sports (7, 11–13, 27, 28). In this study, we therefore aim to give a systematic overview of German media coverage related to mental illness in high-performance sports during the last decade, with formal as well as content-related aspects of print media articles being taken into account. Research on public stigma in Germany indicates that there is still a gap between individual perceptions of public stigma and personal attitudes towards people with mental illness (29). In light of the persistent yet often unrecognized reluctance towards people with mental illness in Germany, our study provides a basis for further research on the understanding, awareness, and societal perception of mental health and illness (30).

### 1.1 Trends in media coverage of mental illness in the general population

Research on media coverage of mental illnesses illustrates that the general picture of people with mental illness displayed by the media is frequently inaccurate and of lay understanding (4, 31). Within media reports, mental illness is regularly associated with violence, aggressiveness, unpredictability and social incompetence, which in turn might further reinforce negative attitudes towards those affected by mental illness within the general population (21, 22, 31, 32). In particular, media sources pertaining to the tabloid press have been found to contain more placative and metaphoric descriptions concerning people affected by mental disorders than the broadsheet press (33, 34). Örnebring and Jönsson (35) described the way tabloid media reports news and stories in general as simplified and personalized, focusing particularly on scandals, drama, and entertainment [see also (34, 36)].

Over the past decade, however, there has been a general shift in media coverage of mental illness in the general population, with media-related research indicating a reduction in inappropriate and stigmatizing language in the broadsheet press (34). This finding stands in line with the recent establishment of guidelines for responsible journalism in a variety of countries, which aim to implement attentive storytelling concerning mental health issues in the media (2, 37–41). In particular, the portrayal of death by suicide has been central to recent media-related studies, as “misinformation, such as […] offering simplistic, monocausal explanations that imply suicide is a solution to immediate trigger” might lead to imitation effects by vulnerable people who identify with the individual exhibiting suicidality [(37), p. 48]. Other potentially harmful features in reporting about suicide were defined as detailed descriptions of the suicide method and photos of the individual deceased, as well as placing the story on the front page or mentioning the method exhibiting suicide in the article's headline (37, 41). Numerous studies have already demonstrated the positive influence considerate and responsible media reporting could achieve, outlining effects related to prevention and help-seeking within the general population (4, 5, 11, 14, 28, 34). Further, media studies have hinted at the important role prominent figures like elite athletes could play for initiating an open and de-stigmatizing discourse about mental illness (13, 14, 42, 43).

### 1.2 Trends in media coverage of mental illness in elite athletes

However, in accordance with the prevalent “winning-at-any-cost”-mentality in high-performance sports, the media traditionally used to portray professional athletes as heroes who sacrifice their bodies for the team and are admired for their fearlessness and courage (1, 2, 43, 44). This dominating picture of *hypermasculinity*, which is deeply embedded within the elite sports system, might hinder affected athletes from discussing their mental health openly and consequently promotes a culture of silence (1, 12, 45, 46).

If the conventional image of the perfect and invulnerable athlete is repeatedly perpetuated in the media, perceptions of mental illness in high-performance sports as well as the risks thereof may be distorted (11). In this context, Teismann et al. (47) examined the media's reporting on the death by suicide of the popular German goalkeeper Robert Enke in November 2009, who kept his longstanding depression private to protect his career. Robert Enke's death by suicide led to intensified but mostly highly inappropriate media reporting. Media reports tended to picture Enke's death as the outcome of a tough and tragic battle, reinforcing the traditional belief in athletes' valor and resilience instead of pointing out alternative approaches to address mental health issues. Particularly tabloid papers tended to place more value on heroizing and sensationalizing reportage than on providing psychoeducational references for people affected (47).

In the aftermath of Enke's death by suicide, not only inappropriate media coverage was criticized, but also the culture of self-sacrifice within the world of high-performance sports (2, 48). As a result, an international discussion has been initiated that questions this culture and rather encourages open conversations about mental health struggles among elite athletes (3, 25, 26, 48). The rise of mental health conversation has also significantly shaped the portrayal of elite athletes and their struggles with mental health in mass media coverage within the last decade (11, 26, 48). Recent studies have shown that athletes who disclose their personal experiences with mental health problems to the public are meanwhile viewed as courageous and mentally strong, embodying role models to look up to (5, 8, 11, 13). For instance, after publicly prioritizing their mental health over athletic success, the female sport stars Naomi Osaka and Simone Biles were highly praised and rewarded by the media (9, 10, 13). Portrayed as strong and brave personalities in the media, these “spokespeople” have become advocates of mental health in the fight against stigmatization (13). Reinforcing the fact that mental illness can affect anyone, media depictions of elite athletes' struggles with their mental health might also carry significant potential for enhancing public understanding of mental illness and its treatment (4, 5, 12, 13, 49).

Overall, the depiction of sensitive topics like mental health and mental illness in the media has just begun to be explored. The specific context of high-performance sports presents a fertile setting for examining media coverage and its effects, as elite athletes are predominantly recognized by the public via their media portrayal (11). Previous research on media portrayals of mental illness has mainly focused on the general population rather than examining depictions of specific social subgroups such as elite athletes (22, 31–33). Looking at media coverage from a broader perspective that extends beyond single events or incidents such as athletes' public disclosures of their mental illness, we sought to develop a general understanding of how the German print media covered mental illness in elite athletes, particularly in light of the recent increase in mental health conversations.

### 1.3 Purpose of the study

Examining the media landscape has been proven to serve as an established method for appraising “the national dialogue around societal issues”, as McGinty et al. (42, p. 1121) pointed out. Thus, the purpose of this media analysis was to give a systematic overview of German print media reportage about elite athletes' mental illness since 2010. In this regard, we aimed to (1) examine if media coverage of mental illness in high-performance sports has changed over time considering content-related (e.g., reported mental disorders, central idea of the article) as well as formal aspects (e.g., article genre, elements of responsible journalism) of media reporting. Further, we were interested (2) in differences between the quality broadsheet press and the tabloid press in Germany. This study is the first to give a systematic overview on media reporting of elite athletes' mental illness over a time period of more than decade. Therewith, the current study expands previous media-related studies examining the portrayal of specific incidents related to mental health issues among elite athletes.

## 2 Method

### 2.1 Data collection

The media analysis comprises articles on mental illnesses of elite athletes published between January 2010 and July 2023 in German newspapers and magazines. The time frame chosen complied with the death by suicide of Robert Enke in November 2009, initiating a rise of mental health awareness within German high-performance sports (26, 48). Our choice for data sources was based on readership and popularity (50), resulting in seven sources identified as traditional broadsheet media (*Frankfurter Allgemeine Zeitung*; *Süddeutsche Zeitung; Die Zeit*; *Die Welt*; *Der Spiegel*; *Focus*; *taz*) and four sources assigned to the tabloid media (*BILD*; *SportBILD*; *BUNTE; Stern*). Newspapers and magazines were identified as pertaining to the broadsheet or tabloid press dependent on their quality of reporting, language, style, diversity, and sources (51). To achieve an equal distribution, further magazines with great popularity pertaining to the tabloid press (*Men's health, Women's health, Fit for Fun, Gala*) were screened but deemed as irrelevant, as they did not report on any content related to our research topic. The estimated readership per issue of the included print media sources ranged from 0.34 million (*taz*) to 10.39 million (*BILD*) in 2010 and from 0.3 million (*taz*) to 6.2 million (*BILD*) in 2022. The search for data sources was limited to the German press landscape, excluding the area of social media. Social media presents a platform of multidirectional communication without identifiable positions of sender and receiver. Consequently, the area of social media did not comply with our aim to analyze depictions of elite athletes' mental illness originating from media institutions.

The search for articles was conducted using three different databases. The international online portal LexisNexis, which provides exact textual replica of published print media articles worldwide, contained nine of the pre-selected newspaper and magazine sources. Two of the traditional broadsheet sources (namely, *Frankfurter Allgemeine Zeitung* (*F.A.Z.*) and *Süddeutsche Zeitung* (*SZ*)) were not included within the LexisNexis database but have established their own online libraries. The strings used to conduct the systematic search were developed by screening related academic work on media presentations of mental illness in elite athletes, as well as published print media articles within the German press. Using Boolean operators, two sets of search terms were combined. To cover a wide range of discussed mental health issues in the media, the first string contained general terms like “mental illness*” or “psychiatric disorder*” as well as specific diagnoses, resulting in a set of 45 search terms. To restrict the search to the specific setting of high-performance sports, the second string included terms related to high-performance sports and contained 11 search terms (e.g., “Olympia”, “high-performance sports”, “athlete”; see Supplementary Table S1 for the complete search string). As the online libraries of the *F.A.Z.* and *SZ* did not allow to conduct a search using Boolean operators, we limited the electronic search to the “Sports” section of each newspaper and applied only the first string of search terms. In total, the systematic search resulted in 17.300 print media articles.

### 2.2 Screening process and final sample

The first author and two trained research assistants screened the title and *highlight* (often consisting of two to four sentences summarizing the articles' main focus) of all 17.300 print media articles, before they read the full texts of the remaining 1,006 articles. For inclusion, print media articles were required to (1) focus on the topic of mental illness of elite athletes (defined as reporting on the mental illness of a specific athlete, mental health issues within high-performance sports in general, specific events or experiences during the athletic career associated with mental health problems, or potential consequences of mental illness on performance, the team, everyday life or the athletic career) and (2) contain more than 75 words. Articles were excluded when they (1) used mental illness-related terms solely in a non-clinical or metaphorical manner (e.g., “…current restrictions resemble a winter depression for passionate skiers…”), (2) mentioned mental illness only peripherally as additional information for the reader, and (3) were classified as “letters to the editor” written by readers. The full-text screening resulted in a final sample of 699 print media articles that were included in further analyses.

### 2.3 Quantitative content analysis

As we aimed to describe selected characteristics of the articles in a systematic and intersubjective manner, we conducted a quantitative content analysis (52) encompassing deductive as well as inductive elements (52, 53).

#### 2.3.1 Deductive and inductive coding

To assess content-related as well as formal aspects of media coverage depicting mental illness, we developed a structured coding scheme based on previous research findings [cf (13, 18, 33, 39, 42, 52, 54)] and inductively derived categories. The final coding scheme consisted of eight elements and 78 outcome items [see Table 1; for more detailed information, see Supplementary Table S2]. Articles were coded for (1) year of publication; (2) type of press (broadsheet or tabloid); (3) article genre (news, feature, interview, comment/column; see Table 1 for their definition); (4) reported mental disorder (in order to explore the media's attention and knowledge regarding specific disorders); (5) central thematic focus, (6) inclusion of elements of responsible journalism (i.e., providing information on helpline-seeking; perspectives of a mental health expert; use of statistics; using appropriate language) (18, 40, 41); (7) quotes from different sources, and (8) perspectives on the high-performance sports system (either criticizing the high-performance sports system or advocating for changes in the sports system with regard to elite athletes' mental health issues).

**Table 1:** Variables covering formal and content-related aspects.

Variable	Description
Formal aspects	
Time period 1/2	Captures the year of publication, subsequently divided into period 1 (January 2010 until December 2016) and period 2 (January 2017 until July 2023).
Type of press	Determines whether articles pertain to the tabloid or broadsheet press.
Article genre	Refers to the specific genre of the article based on its style, purpose and content (*news, feature, interview, comment/column*). News articles were defined as informational and factual texts. Feature articles involve a shift in perspective, allowing the author to move from a factual, objective narrative to subjective insights and a personal focus. Articles classified as *Comment* or *Column* reflect the author's personal attitudes and/or experiences and are therefore significantly shaped by personal opinion (55).
Elements of responsible journalism	Refers to the considerate and accurate dissemination of information concerning mental illness by journalists or media outlets. Responsible reporting is represented by four outcome items: help-seeking information; statistics; appropriate language and the perspective of a mental health expert (defined as an individual with an educational background in medicine, science, health, or psychology who provides general public health information without offering professional opinions or diagnoses regarding the mental health of public figures [in accordance with the *Goldwater Rule* (56)].
Content-related aspects	
Reported mental disorder	Captures the type of mental disorder(s) discussed in the article or the absence thereof (i.e., if no specific diagnosis is mentioned).
Central thematic focus of the article	Describes the central thematic focus of the article (e.g., death by suicide of a famous athlete; end of career; disclosure; treatment/hospitalization).
Sources of comments and quotations	Captures direct quotes from (1) the person affected, (2) the social or professional environment of the person affected, (3) a mental health expert defined as persons with an educational background in medicine, science, healthcare, or psychology.
Perspectives on the high-performance sports system	Captures either (1) any critical statement, quote, or expression, indicating disapproval or concerns related to the established framework of the high-performance sports system or (2) describe any positive change or development initiated for preventing mental illness as well as support for those affected (e.g., launch of charities).

To increase transparency and consistency of coding, a detailed codebook was developed and tested by two coders (i.e., the first author and one trained research assistant) with 5% of the final sample. Some coding criteria were revised and refined to facilitate and clarify the identification of elements. For instance, the initial element “perspectives on the high-performance sports system” was revised in such a way that only statements that either clearly criticized the high-performance sports system or advocated for changes in the sports system with regard to elite athletes' mental health were coded. Intercoder reliability was tested with a random sample of 10% of the articles in each newspaper/magazine (*n* = 70 articles) that were coded by both coders independently as suggested by Neuendorf (57). To determine intercoder reliability, we used Cohen's kappa (*κ*) for those variables with multiple response options and applied the McNemar test to binary variables (58, 59). The three items with multiple response options met acceptable to substantial intercoder reliability (i.e., *κ*_central thematic focus_ = .824; *κ*_article genre_ = .633; *κ*_diagnosis_ = .62). McNemar test results ranged from *p* = .625 to *p* = 1.000, which is also considered as acceptable to substantial intercoder reliability (13, 42, 58). As a result, the remaining 90% of articles were coded by only one of the two coders.

#### 2.3.2 Statistical analysis

Statistical analyses were conducted using SPSS Version 28 and Microsoft Excel. To provide a systematic overview of the German media coverage of elite athletes' mental health issues, descriptive statistics were calculated, determining frequencies and proportions of coded elements on an overall item level. To explore changes over time, the investigation period was split into time period 1 (January 2010–December 2016) and time period 2 (January 2017–July 2023) as recommended and applied by previous media studies to improve comparability and enhance practicability [cf. (33, 42)]. Further, those elements offering more than one response option (i.e., “article genre”; “reported mental disorder”; “central thematic focus of the article” and “elements of responsible journalism”) were converted into dichotomous yes/no variables.

Univariate analyses (*χ*^2^ tests) were conducted to assess significant associations between the independent variables (*time period*; *type of press*) and the categorical elements (“article genre”; “reported mental disorder”; “central thematic focus of the article”; “elements of responsible journalism”; “perspectives on the high-performance sports system” and “sources of comments and quotations”). Further, binary logistic regression was conducted to calculate the odds a categorical element was influenced by the predictors *time period* (2010–2016) and *type of press* (tabloid vs. broadsheet)*.* A significance level of *α* < .05 was applied to all statistical tests.

## 3 Results

The final data sample (*n* = 699) contained 609 articles pertaining to the broadsheet press (F.A.Z., *n* = 79; SZ, *n* = 222; Die Zeit, *n* = 14; Die Welt, *n* = 75; Der Spiegel, *n* = 152; Focus, *n* = 16; taz, *n* = 51), and 90 articles from the tabloid press (BILD, *n* = 16; SportBILD, *n* = 42; Stern, *n* = 23; BUNTE, *n* = 9). Considering the dominance of broadsheet sources included in our study, data analyses were adapted to the uneven distribution. The average word count for all included articles was 837 (Min = 94, Max = 5,932). There was no statistically significant difference in the average word count between print media articles pertaining to the broadsheet press (*M* = 817, *SD* = 676) and the tabloid press (*M* = 963, *SD* = 754); *t*(697) = −1.87, *p* = .061.

### 3.1 Time trends within the amount of media coverage

**Figure 1:**
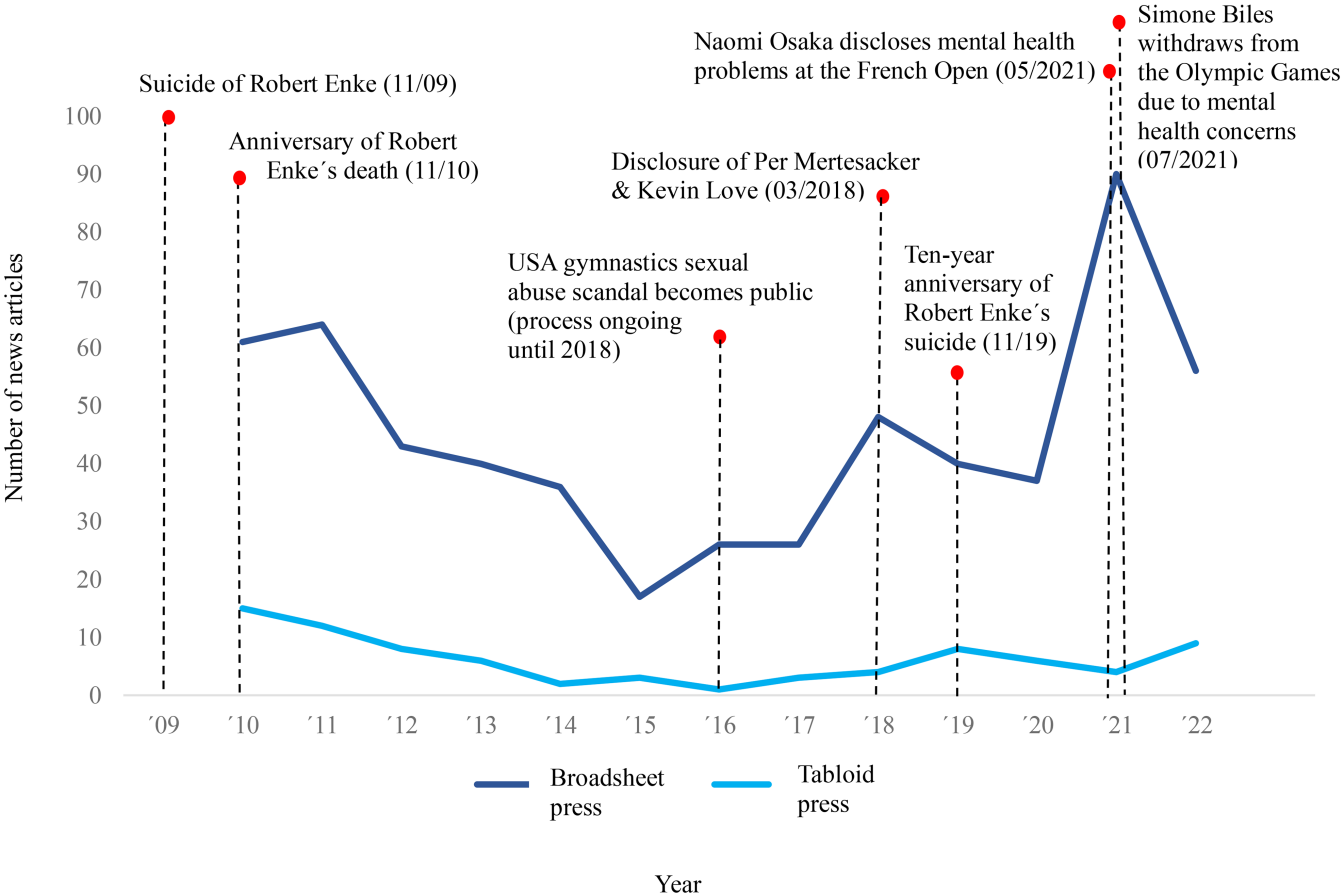
Volume of German news coverage of elite athletes' mental health issues in different types of press and specific incidents within the investigation period.

The volume of print media articles covering the mental illness of elite athletes was found to be highly associated with specific events and incidents (see Figure 1). Peaks of media reporting could be observed concerning tragic incidents (e.g., the death by suicide of goalkeeper Robert Enke in 2009), scandals being uncovered within the world of high-performance sports (e.g., sexual abuse of under-age gymnasts within the association *USA gymnastics*, revealed in 2016), or disclosures of famous personalities (e.g., the soccer player Per Mertesacker talking openly about the pressure he experienced as unbearable in professional soccer in the context of his retirement in 2018).

### 3.2 Article genre

The most common article genre was news (*n* = 393), followed by features (*n* = 132), interviews (*n* = 109), and comments/columns (*n* = 65). While changes over time could not be detected, a significant difference was found between the broadsheet and the tabloid press for almost every article genre, except for features (for detailed descriptive statistics see Tables 4, 5). The results indicate that news (*n* = 393, *β* = −.701, odds ratio = .498, 95% CI: 0.316–.778, *p* = .002) as well as comments/columns (*n* = 65, *β* = −1.190, odds ratio = .304, 95% CI: .093–.991, *p* = .048) were more likely to appear in the broadsheet press than in the tabloid media. In contrast, the genre interview showed a strong association with tabloid papers (*X*^2^ = 20.608; *p* = .001), with a 3.4 times higher likelihood for interviews to appear in the tabloid press than in the broadsheet press (*β* = 1.210, odds ratio = 3.354, 95% CI: 2.039–5.519, *p* = <.001).

### 3.3 Central thematic focus of the article

The three most frequent topics appearing in the included print media articles were “current status” (*n* = 132, 19%), “disclosure” (*n* = 108, 16%) and “criticism of the system” (*n* = 91, 13%). Articles were coded as “current status” if the print media article reported on the athlete's mental health condition in the context of future or past performances and competitions. Articles whose central thematic focus was coded as “disclosure” narrated the affected athlete's act of *revealing* a mental health condition and, consequently, sharing details or facts that were previously unknown or private. Articles were coded as “criticism of the system” if they primarily focused on disapproval or dissatisfaction with the high-performance sports system, its structure or embedded values and beliefs. Often, areas that needed improvement or reform were highlighted within these articles (for further information on the different central thematic focuses of the articles see Supplementary Table S3).

Looking at changes over time, four of the 14 predefined outcome items in the category of thematic focus differed significantly between the first and the second investigation period (for a detailed descriptive overview see Table 2). Articles pertaining to the first time period (2010–2016) reported much more frequently about deaths by suicide than articles published later than 2017 (*β* = −1.711, odds ratio = .181, 95% CI: .092–.354, *p* = <.001). The clear predominance compared to the second time period might be explained by the death by suicide of Robert Enke, which elicited intensified media reporting in Germany.

**Table 2:** Central thematic focus of articles according to the year published and the type of press.

	2010–2016 (*N* = 334)	2017–2023 (*N* = 365)	Chi square		Broadsheet press	Tabloid press	Chi square	
	No. (%)	No. (%)	Value	*p*	No. (%)	No. (%)	Value	*p*
Central idea of the article								
Current status	66 (20)	66 (19)	.443	.506	116 (19)	16 (18)	.105	.746
End of career	15 (5)	22 (6)	.821	.365	33 (5)	4 (4)	.148	.700
Portrait of life	30 (9)	33 (9)	.001	.978	49 (8)	14 (16)	5.392	.020*
Abuse	3 (1)	34 (9)	24.647	<.001**	34 (6)	3 (3)	.792	.374
Disclosure	50 (15)	58 (16)	.019	.891	96 (16)	12 (13)	.269	.604
Death by Suicide	49 (15)	11 (3)	30.202	<.001**	47 (8)	13 (14)	4.522	.033*
Therapy	3 (1)	3 (1)	.012	.913	6 (1)	0 (0)	.894	.344
Injury	2 (1)	2 (1)	.301	.583	3 (1)	2 (2)	3.303	.069
Deviating behavior	22 (7)	25 (7)	.019	.890	44 (7)	3 (3)	1.893	.169
Neurological disorder	9 (3)	2 (1)	5.189	.023*	9 (2)	2 (2)	.281	.596
Hospitalization	7 (2)	0 (0)	7.727	.005*	5 (1)	2 (2)	1.553	.213
Criticism of the system	42 (13)	49 (13)	.111	.739	80 (13)	11 (12)	.058	.810
Description of the system	9 (3)	15 (4)	1.053	.305	24 (4)	0 (0)	3.673	.055
Change of the system	26 (8)	45 (12)	3.947	.047*	63 (10)	9 (11)	.182	.670

* *p* < .05.

** *p* < .001.

A further reduction from the first to the second time period was detected regarding the topic of “neurological disorders” (*β* = −1.615, odds ratio = .199, 95% CI: .043–.928, *p* = .040). The difference in the number of articles can probably be explained by the discovery of an association between repeated concussions due to head traumata and the occurrence of mental health impairments (e.g., depression, death by suicide, behavior change) within the first time period (60). This discovery brought great attention to the risks of participating in contact sports, particularly in the US, and led to intensified research and media coverage (61).

The central thematic foci, which were found to appear significantly more often in the second investigation period dealt with (sexual) abuse (*β* = 2.428, odds ratio = 11.333, 95% CI: 3.477–27.263, *p* = <.001) and changes of the system (*β* = .510, odds ratio = 1.666, 95% CI: 1.003–2.767, *p* = .049). The strong association of the topic of sexual abuse with the later investigation period (*X*^2^ = 24.647, *p* = <.001) can likely be attributed to the sexual abuse scandal within the association *USA gymnastics*, which provoked intense controversy about safety in sports and led to international media attention. Enhanced awareness is also part of the outcome item “change of the system”, where print media articles reported on initiated improvements within the high-performance sports system (e.g., the creation of foundations for athletes affected by mental health problems).

With regard to press type, some differences could be observed. While in broadsheet papers only 8% of all included articles were coded as “portrait of life”, the outcome item was found to be significantly associated with the tabloid press (*X*^2^ = 5.392, *p* = .029). “Portraits of life” usually provided information about an athlete's childhood and family situation, the beginning of her/his sportive career as well as failures and successes, often highlighting personal issues that jeopardized or strengthened their mental health. Logistic regression revealed that portraits of life appeared twice as often in the tabloid media as in the broadsheet media (*β* = .744, odds ratio = 2.105, 95% CI: 1.110–3.994, *p* = .023).

Further, the share of articles reporting on “death by suicide” was significantly higher in the tabloid press (14% of all articles, *n* = 13) than in the broadsheet press (8% of all articles, *n* = 47) (*X*^2^ = 4.522, *p* = .043). The likelihood for “death by suicide” representing the focus of an article was two times higher in tabloid papers than for articles originating from broadsheet media (*β* = .702, odds ratio = 2.019, 95% CI: 1.045–3.901, *p* = .037). None of the remaining outcome items revealed a significant association with the *type of press*.

### 3.4 Reported mental disorders

The three most common mental disorders reported on within the included print media articles were depression (*n* = 352), burnout (*n* = 164) and substance use disorder (*n* = 99) (for a detailed descriptive overview see Table 3). It is important to mention that these mental disorders were recorded based on the way they were referred to in the articles, not based on established diagnostic criteria.

**Table 3:** Reported mental disorder according to the year published and the type of press.

	2010–2016 (*N* = 334)	2017–2023 (*N* = 365)	Chi square		Broadsheet press	Tabloid press	Chi square	
	No. (%)	No. (%)	Value	*p*	No. (%)	No. (%)	Value	*p*
Reported mental disorder								
Depression	182 (55)	170 (47)	4.371	.037*	302 (50)	50 (56)	1.119	.290
Burnout	111 (33)	53 (15)	34.010	<.001**	135 (22)	29 (32)	4.148	.042*
Panic disorder	6 (2)	17 (5)	4.457	.035*	20 (3)	3 (3)	.000	.983
Substance use disorder	64 (19)	35 (10)	13.146	<.001**	83 (14)	16 (18)	1.110	.292
Behavioral addiction	16 (5)	5 (1)	6.968	.008*	14 (2)	7 (8)	6.055	.014*
Anxiety disorder	19 (6)	27 (7)	.828	.363	38 (6)	8 (9)	.895	.344
Bipolar disorder	8 (2)	6 (2)	.502	.479	14 (2)	0 (0)	2.111	.146
Without diagnosis	27 (8)	99 (27)	42.562	<.001**	119 (20)	7 (8)	8.750	.003*
Obsessive-compulsive disorder	3 (1)	2 (1)	.301	.583	3 (1)	2 (2)	3.303	.069
ADHD	2 (1)	2 (1)	.008	.929	3 (1)	1 (1)	.527	.468
PTSD	4 (1)	8 (2)	1.022	.312	12 (2)	0 (0)	1.804	.179
Eating disorder	14 (4)	36 (10)	13.763	<.001**	43 (7)	7 (8)	.001	.980

* *p* < .05.

** *p* < .001.

The mental disorders mentioned in print media articles changed substantially throughout the entire investigation period. Depression and burnout decreased significantly in their media appearance within the second time period (2017–2023). The likelihood of depression being mentioned in a print media article was found to be significantly lower in more recent years (*β* = −.317, odds ratio = .728, 95% CI: .541–.981, *p* = .037). The decline of articles mentioning burnout was even more pronounced (*β* = −1.075, odds ratio = 0.341, 95% CI: 0.236–0.494, *p* = <.001), with the number of articles reporting on burnout of elite athletes in the first time period (*n* = 111, 33%) being reduced by more than half after 2016 (*n* = 53, 15%). A similar decline was found for the diagnosis of substance use disorder (*β* = −.804, odds ratio = .447, 95% CI: .288–.696, *p* = <.001) and behavioral addictions (e.g., gambling addiction) (*n* = 6, *β* = −1.284, odds ratio = .277, 95% CI: .100–.764, *p* = .013), which, however, constituted only a minor subset of all reported mental disorders.

The opposite time trend was observed concerning panic disorders (*β* = .979, odds ratio = 2.662, 95% CI: 1.037–6.835, *p* = .042) and eating disorders (*β* = .908, odds ratio = 2.480, 95% CI: 1.517–4.055, *p* = <.001). The greatest increase over time was detected for articles reporting no diagnoses at all (*n* = 126). While in the first period, only 8% of the articles did not mention a specific mental disorder, the amount has more than tripled within recent years (27%). Performing logistic regression, a 4.2 times higher likelihood was identified for the second period to include articles without mentioning any specific disorders (*β* = 1.439, odds ratio = 4.218, 95% CI: 2.673–6.656, *p* = <.001), and rather referring to mental health problems in general [e.g., “The 2017 mass start world champion took a voluntary break at the World Cup in Oberhof in January this year, feeling physically and mentally exhausted.”, see (62)].

Concerning the type of press, less differences were detected. Burnout was reported on more frequently in tabloid papers than in the broadsheet media (*β* = .512, odds ratio = 1.669, 95% CI: 1.031–2.702, *p* = .037). Logistic regression revealed similar results concerning behavioral addictions, presenting a 3.6 times higher likelihood to appear in tabloid papers than in the broadsheet press (*β* = 1.275, odds ratio = 3.578, 95% CI: 1.404–9.123, *p* = .008). In contrast, articles without mentioning a specific mental disorder were detected to mostly pertain to the broadsheet press (*β* = −1.060, odds ratio = .347, 95% CI: .156–.769, *p* = .009). Logistic regression revealed that the likelihood of a mental disorder being mentioned was almost three times higher for tabloid media (*β* = 1.078, odds ratio = 2.940, 95% CI: 1.325–6.521, *p* = .008). To illustrate, 92% of articles published in tabloid papers included a specific diagnosis in their reporting (compared to 80% in the broadsheet press).

### 3.5 Sources of comments and quotations

Overall, the most frequent source to be cited directly in the print media articles throughout the entire investigation period as well as in both types of press was the affected athlete him- or herself (*n* = 548, 78%) [e.g., “‘It's okay not to be okay,’ Osaka recently wrote in a Time magazine article. And Biles said in Tokyo that athletes are ‘not just athletes—at the end of the day, we are human beings.’”, see (63)]. The person affected became an element of even greater attention within the second time period (see Table 4) with 82% of all articles including direct quotations by the affected athlete (*β* = 436, odds ratio = 1.546, 95% CI: 1.076–1.045, *p* = .019).

**Table 4:** Results of chi-square tests and logistic regression analyses for both time periods.

	2010–2016 No. (%)	2017–2023 No. (%)	Chi square (df = 1)	*p*	*β*	Odds ratio (95% CI) for period 1/2	*p*
Article genre							
News	177 (53)	216 (59)	2.711	.100	.251	1.286 (0.953–1.735)	.100
Feature	71 (21)	61 (17)	2.350	.125	−.297	.743 (0.508–1.087)	.126
Interview	55 (17)	54 (15)	.370	.543	−.127	.881 (0.585–1.326)	.543
Comment, Column	31 (9)	34 (9)	.000	.988	.004	1.004 (.602–1.674)	.988
Sources of comments and quotations							
Person affected by a mental illness	249 (75)	299 (82)	5.590	.018*	.436	1.546 (1.076–2.223)	.019*
Mental health expert	62 (19)	50 (14)	3.066	.080	−.362	.696 (.464–1.045)	.081
Family/friends/teammates/collegues	217 (65)	210 (58)	4.066	.044*	−.314	.730 (.538 –.992)	.044*
Elements of responsible journalism							
Helpline information	76 (23)	68 (19)	1.812	.178	−.252	.777 (.538–1.122)	.179
Statistics	64 (19)	48 (13)	4.686	.030*	−.448	.639 (.425 –.961)	.031*
Perspective of a mental health expert	85 (25)	71 (20)	3.616	.057	−.346	.707 (.495–1.011)	.058
Appropriate language	262 (79)	334 (92)	24.125	<.001**	1.085	2.961 (1.886–4.648)	<.001**
0 Elements	35 (11)	17 (5)	8.701	.003*	−.874	.417 (.229 –.760)	.004*
1 out of 4 Elements	177 (53)	233 (64)	8.463	.004*	.448	1.566 (1.157–2.120)	.004*
2 out of 4 Elements	66 (20)	70 (20)	.038	.846	−.037	.964 (.662–1.402)	.846
3 out of 4 Elements	43 (13)	31 (9)	3.540	.060	−.465	.628 (.386–1.023)	.062
4 out of 4 Elements	11 (3)	14 (4)	.149	.699	.158	1.171 (.524–2.617)	.700
Perspectives on the high-performance sports system							
Criticism of the system	157 (47)	198 (54)	3.661	.056	.290	1.337 (.993–1.800)	.056
Change of the system	111 (33)	153 (42)	5.613	.018*	.371	1.450 (1.065–1.974)	.018*

* *p* < .05.

** *p* < .001.

Quotes of the (social) environment—including for instance coaches, teammates, friends, or family—were found to have a slightly lower chance to appear in articles published later than 2016, decreasing from 65% in time period 1 to 58% in time period 2 (*β* = −.314, odds ratio = .730, 95% CI: .538–.992, *p* = .044). A mental health expert was only cited in 16% (*n* = 112) of articles, with no significant change over time. Regarding the type of press, no significant differences could be detected for any of the three sources (see Table 5).

**Table 5:** Results of chi-square tests and logistic regression analyses for type of press.

	Broadsheet press No. (%)	Tabloid press No. (%)	Chi square (df = 1)	*p*	*β*	Odds ratio (95% CI) for type of press	*p*
Article genre							
News	356 (59)	37 (41)	9.508	.002*	−.701	.496 (.316–.778)	.002*
Feature	112 (18)	20 (22)	.725	.394	.237	1.268 (.741–2.170)	.387
Interview	79 (13)	30 (33)	20.608	<.001**	1.210	3.354 (2.039–5.519)	<.001**
Comment, Column	62 (10)	3 (3)	5.474	.019*	−1.190	.304(.093–.991)	.048*
Sources of comments and quotations							
Person affected by a mental illness	474 (78)	74 (82)	.928	.335	.276	1.317 (.743–2.337)	.346
Mental health expert	98 (16)	14 (16)	.017	.897	−.040	.961 (.522–1.767)	.897
Family/friends/teammates/collegues	371 (61)	56 (62)	.056	.813	.055	1.057 (.670–1.667)	.813
Elements of responsible journalism							
Helpline information	117 (19)	27 (30)	.152	.023*	.589	1.802 (1.100–2.953)	.019*
Statistics	99 (16)	13 (14)	.196	.658	−.140	.870 (.465–1.626)	.662
Perspective of a mental health expert	135 (22)	21 (23)	.061	.805	.066	1.069 (.632–1.806)	.804
Appropriate language	532 (87)	64 (71)	13.983	<.001**	−1.032	.356 (.213–.596)	<.001**
0 Elements	41 (7)	11 (12)	2.995	.084	.657	1.929 (.952–3.908)	.068
1 out of 4 Elements	365 (60)	45 (50)	3.150	.076	−.403	.668 (.429–1.042)	.075
2 out of 4 Elements	112 (18)	24 (27)	3.200	.074	.478	1.614 (.969–2.688)	.066
3 out of 4 Elements	66 (11)	8 (9)	.328	.567	−.220	.803 (.372–1.733)	.576
4 out of 4 Elements	23 (4)	2 (2)	.621	.431	−.546	.578 (.134–2.499)	.464
Perspectives on the high-performance sports system							
Criticism of the system	307 (50)	48 (53)	.268	.605	.117	1.124 (.721–1.725)	.605
Change of the system	223 (37)	41 (46)	2.616	.106	.370	1.448 (.927–2.263)	.104

* *p* < .05.

** *p* < .001.

### 3.6 Elements of responsible journalism

Most media guidelines for reporting on mental illness discuss four elements of responsible journalism, including the integration of statistics, the use of appropriate language, the provison of help-seeking information, and the inclusion of an expert opinion (18, 40, 41).

Concerning the *time period* as predictor, the integration of statistics in media articles [e.g., “In a study conducted by Deutsche Sporthilfe (German Sports Aid Foundation), more than a thousand top athletes were surveyed. […] One in five suffers from occasional depression or burnout, one in ten has an eating disorder.”, see (64)] was significantly less likely in the second investigation period, decreasing from being mentioned in 19% of the articles to 13% (*β* = −.448, odds ratio .639, 95% CI: .425–.961, *p* = .031). In contrast, a highly significant association could be determined between the second time period and the outcome item “appropriate language” (*X*^2^ = 24.125, *p* = <.001). Articles used appropriate language with an almost three times higher likelihood between 2017 and July 2023 (*β* = 1.085, odds ratio = 2.961, 95% CI: 1.886–4.648, *p* = <.001). The use of the remaining two elements of responsible journalism (i.e., helpline information, inclusion of an opinion of a mental health expert) did not differ significantly between both time periods (see Table 4).

When focusing on the proportion of implemented elements of responsible journalism, logistic regression revealed a significantly lower likelihood for articles to contain no element of responsible journalism within the second time period (*β* = −.874, odds ratio = .417, 95% CI: .229–.760, *p* = .004). Similarly, a highly significant association could be detected between the appearance of one element of responsible journalism and the second time period (*X*^2^ = 8.463, *p* = .004), with 64% of all analyzed articles between January 2017 and July 2023 containing either helpline information, statistics, appropriate language, or the opinion of a mental health expert (*β* = .448, odds ratio = 1.566, 95% CI: 1.157–2.120, *p* = .004).

For the predictor *type of press,* two out of the four elements of responsible journalism reached levels of significance: The item “helpline information” had a significantly higher likelihood to appear in the tabloid press than in broadsheet articles (*β* = .589, odds ratio = 1.802, 95% CI: 1.100–2.953, *p* = .019). In contrast, the use of appropriate language was clearly associated with the broadsheet press (*X*^2^ = 13.983, *p* = <.001). Here, 87% of articles were written responsibly (*β* = −1.032, odds ratio = 0.356, 95% CI: .213–.596, *p* = <.001), in contrast to 71% of articles in the tabloid press. Examining the influence of the type of press on the proportion of implemented elements of responsible journalism revealed no significant differences (see Table 5).

### 3.7 Perspectives on the high-performance sports system

Over time, we observed a nonsignificant increase (*β* = .290, odds ratio = 1.337, 95% CI: .993–1.800, *p* = .056) of print media articles including statements that criticized the high-performance sports system, such as “Ewald Lienen talks about the revelations of his former protégé Per Mertesacker and the reasons why the Bundesliga makes you sick.” (65). In contrast, statements that described a “change of the system” (i.e., reporting on enhanced mental health awareness in high-performance sports, or drawing attention to implemented or planned initiatives to prevent or reduce mental illness among members of the sports system) were significantly more likely to appear in articles within the second time period (*β* = .371; odds ratio = 1.450; 95% CI: 1.065–1.974; *p* = .018).

Regarding the *type of press*, no significant influence on the occurrence of criticizing statements (*β* = 0.117, odds ratio = 1.124, 95% CI: .721–1.725, *p* = .605), nor on statements referring to a change of the system (*β* = .370, odds ratio = 1.448, 95% CI: .927–2.263, *p* = .104) could be detected.

## 4 Discussion

Our analysis is the first to our knowledge that examines more than one decade (2010–2023) of print media reporting about elite athletes' mental illness. Previous studies have assessed changes in media coverage of mental illness in elite athletes often in reference to specific events or incidents, such as examining media coverage before and after Michael Phelps ended his career at the 2016 Olympics in Rio) (4, 8). The increasing attention that the topic of mental health receives in media-related research can be attributed to the growing mental health awareness partly guided and supported by elite athletes who talk openly about their mental health struggles in the media (11). In line with the gradual shift towards enhanced mental health awareness within the world of high-performance sports, our systematic analysis of the German print media landscape indicated that the understanding and openness towards the sensitive topic of mental illness have increased throughout the entire investigation period as well as across different press types.

### 4.1 Discussion of time trends

In prior research, mass media content has been identified as an indicator of public discourse, thus representing public opinion and changes thereof (19, 33, 39, 42). Examining the media landscape over time, our results suggest that single incidents like the death by suicide of a popular athlete have the power to strongly influence the amount and prioritization of overall reporting on mental illness.

Focusing on the central thematic idea of the included print media articles, our findings show that recently published articles tended to report more frequently on topics that question the glamorous appearance of the high-performance sports system and took a more critical stance towards high-performance sports. Conventionally, media coverage used to reflect values typical of the culture of risk deeply embedded in the sports system (such as the win-at-all-costs mentality) and celebrated athletes as invincible heroes (8, 12, 15, 43). In this regard, Sanderson et al. (43) designated the interaction between sports media and the sports system as a “symbiotic relationship” (p. 8), with the media reinforcing the spectators' imagination of elite athletes as the ideal of human perfection. However, our study revealed that the idealized notion of the high-performance world has recently come under increasing criticism in the media. For instance, in articles covering the well-known abuse scandal occurring within the association *USA gymnastics* revealed in 2016, the media not only highly despised the deliberate concealment of those responsible but also criticized prevailing hierarchies and dependencies in the world of high-performance sports, questioning high-performance sports' heavy emphasis on performance [cf. (66)].

This trend is further reinforced by the increasing implementation of first-person narratives and direct speech in the context of sports media coverage, complementing the predominant focus on just performance. Our findings show that people affected by mental health issues were the source most frequently cited within the included print media articles, specifically within the second investigation period. This finding supports latest research on media coverage, which demonstrates that athletes are nowadays more frequently quoted in news stories related to mental disorders, giving them the chance to speak up for themselves (8, 11–13, 48). Further, as Gwarjanski and Parrott (22) have pointed out, the most effective way to combat stigma related to mental illness is through personal contact with individuals affected (5, 22, 34, 67). In this context, research on health communication and sport showed that also *parasocial contact*, defined as the “one-sided relationship with media personae” (5, p. 149), has the potential to reduce prejudices and sensitize people for mental health (5, 11, 67). The increasing number of athletes talking openly about their mental health struggles in the media therefore carries the potential to reduce mental illness stigma (5, 14, 67). However, in this context, it is also important that the media do not unquestioningly and uncritically present the personal attitudes and opinions of athletes themselves. For instance, the presentation of a celebrity athlete's treatment or lifestyle advice that is mainly based on their own experiences and not on scientific evidence could indeed adversely affect public understanding and, in the longer term, have negative real-world consequences [see also (68)].

Also, mental illnesses were found to be no longer depicted in a sensationalist or stigmatizing manner, as criticized in some studies of the past decade (18, 31, 33, 39). Instead, print media articles tended to handle medical diagnoses more cautiously and neutral. Although mental illnesses are nowadays better researched and understood (4), most articles from the second investigation period did not mention any diagnosis at all. An explanation for this trend could be the rising societal importance of various mental health issues leading to an increasing incorporation of the topic by the media. The example of Simone Biles' withdrawal from the 2021 Tokyo Olympics illustrates the great attention that mental health issues are nowadays receiving in the media (9). By being honest about her need to protect her mental health, Biles was highly praised by the media for her courage while rumors of a possible mental illness were rarely found in articles (9, 10). The growing awareness for confidentiality and privacy rights may offer another explanation for the finding. In this context, new ethical guidelines, such as the *Goldwater Rule* established by the American Psychiatric Association (56), which prohibits psychiatrists from offering a professional diagnosis of public figures without having conducted a personal examination, might also have repercussions on how mental health is addressed in the media.

The rise of mental health awareness was not only evident regarding content-related aspects of print media articles but was also reflected in formal aspects, particularly with regard to responsible journalism. In collaboration with the World Health Organization, specific media guidelines have been established to enhance sensitivity and diligence in the context of journalistic work (2, 37). Our results indicate that the media increasingly report considerately on mental illness of elite athletes, as pointed out by prior media-related research (14, 18, 22, 38, 54). This is particularly reflected in the more frequent use of accurate language in recent years, avoiding metaphoric or dramatizing expressions.

### 4.2 Discussion of press type

Nevertheless, the intention of the media to “entertain” the audience carries the risk of emphasizing certain aspects of a story while minimizing other information and, thereby, shape the perception and salience of a story (22, 43, 69). Caught between fulfilling expectations and educating the public, journalists tend to structure their narratives according to the ideas and attitudes of the respective interest groups their stories are addressed to (8, 15). Our study revealed that there are significant differences between press types in terms of their approach to the topic of mental illness.

Overall, our results suggest that the examined German tabloid media mostly did not meet common professional standards in media coverage (51). Particularly with regard to story content and responsible journalism, our findings confirmed the lack of quality reporting on mental illness within tabloid media as indicated by previous studies (33, 35, 51). It was clearly noticeable that tabloids attached great importance to sensationalizing reports. According to our comparative analysis, tabloid papers tended to present their news and stories more in a lively manner, emphasizing personal fate and focusing preferably on personal experiences than on data and facts (34, 36, 70). To illustrate, the proportion of interviews that by default provide personal insights rather than neutral information was found to be significantly higher in tabloid papers than in the broadsheet press. However, including more first-person narratives (such as interviews) in reporting can also be seen as counteracting stigma. Wahl (71) pointed out that the absence of interviews in the media with people who have experienced mental illness might lead to the assumption that they are “too disordered, too disorganized, too unreliable to speak for themselves” (p. 1598).

In terms of responsible reporting, broadsheet papers were found to report more responsibly on mental illness, particularly with regard to the use of appropriate language. In contrast, tabloid papers seemed to place far more emphasis on the emotional impact of their coverage, by using emotive and placative expressions when portraying people affected. Our results confirm previous findings regarding the increased use of metaphorical and equating descriptors in tabloid media coverage (33, 72, 73). Clement and Foster (33) attributed the use of inappropriate language to the goal of enhancing the entertainment value of news stories. The only exception regarding the predefined elements of responsible journalism was the inclusion of helpline information, which appeared more frequently in tabloid papers. This deviation could be explained by the fact that the sensitive topic of suicide represented the third most common thematic focus in tabloid press articles. According to established media guidelines (40, 41), references to helpline information are obligatory when reporting about death by suicide and similar incidents (e.g., suicidal behavior, attempts to exhibit suicide).

The accurate reporting in broadsheet papers was further demonstrated by a higher sensitivity to the reported mental disorders. Broadsheet articles appeared to be more cautious, focusing less on official diagnoses and more on the wellbeing and current mental health of the athletes concerned. This approach to sensitive issues indicates that broadsheet papers may have been more effective than tabloids in responding to increasing public awareness of mental health. Another possible explanation for reporting on elite athletes' mental health problems in general, even in the absence of a diagnosis, may be a recognition by the broadsheet press of the inherent value of discussing mental health more broadly.

In this context, our findings also show that tabloid papers presented far fewer critical comments in their reportage that questioned embedded values of the high-performance sports system and encouraged critical thinking. Previous research has indeed demonstrated that the tabloid press tends to attract readers by focusing on sensational and dramatic stories, supplemented by private details, thereby neglecting the educational function of the media (35, 70). From the media's point of view, finding a balance between the search for sensational stories and acting with consideration could be a difficult challenge.

### 4.3 Limitations and future research

Applying a systematic search strategy in the LexisNexis database represents a strength of the current study. However, the external appearance of a print media article, its positioning within the printed paper, typeface, and images could not be included in our study, as the databases used did not provide this information. The coding process represented another potential limitation of the current study due to the subjective assessment some outcome items required (such as deciding on the main thematic focus of the article). To counteract this limitation, we developed a detailed codebook that contained intersubjectively comprehensible rules for coding as suggested within similar media-related research (39, 42, 73). Further, both reviewers coded 10% of the sample demonstrating substantial intercoder reliability (39, 74).

In the context of interpretation, it is also important to consider the journalistic perspective when analyzing print media articles. Journalists' own experiences and predispositions may result in possible biases (24, 75). We must consider that media personal might diagnose a mental illness more quickly than necessary to fit their story line and to attract the reader's interest. Also, once an official mental disorder diagnosis has been made, journalists may interpret and present an athlete's behavior differently in future reports. It is therefore important to note that we did not examine the occurrence of mental illnesses in the world of high-performances sports, but its appearance in the media.

To bypass the media's role as gatekeeper of information and to take control of their own narrative, many athletes have recently chosen to share their stories through social media (11–13). Excluding social media from our analysis can be seen as a limitation. With its specific features such as allowing for multidirectional communication and *parasocial contact* (11, 34), social media represents a promising avenue for future media and mental health research within the context of high-performance sports.

Although the perceived taboo of mental illness is increasingly lifted, there is a clear need for future media-related studies to explore the traditional media's and social media's role in this process (11). In this regard, a promising direction for future research could be comparisons across different national contexts, examining how cultural and traditional narratives shape media representations and how the public is influenced by them. To build specifically on our findings and thus complement the broad overview presented in this paper, future qualitative research could also explore print media articles at a more interpretive level, examining the frames in which stories about elite athletes' mental health are embedded.

## 5 Conclusion

Media stories presenting personal fate like the rise and fall of prominent figures like elite athletes will always meet great interest in the public (12–14, 27). To promote critical and independent thinking in society, it is of utmost importance that the media report responsibly on sensitive issues such as mental illness or death by suicide rather than undermining facts with emotion and prurience to capture readers' interest. Overall, the findings of the current study suggest that the media seem to be taking a more critical and responsible approach to the topic of mental illness in high-performance sports. By increasingly giving voice to those affected, the media are demonstrating a greater understanding of the importance and seriousness of the issue. Furthermore, our media analysis shows that the media have begun to distance themselves from the ideology of the sports culture. The media's willingness to question conventional ideals embedded in the sports culture may reflect and further contribute to mental health awareness in society.

## Data Availability

The data analyzed in this study is subject to the following licenses/restrictions: The data included in the manuscript are articles published in newspapers and magazines. Therewith, they are publicly available, however, sometimes behind a paywall. Requests to access these datasets should be directed to Marcia Hapig, marcia.hapig@uni-tuebingen.de.
