# Supplementary material for: Exploring print media coverage of elite athletes’ mental illness between 2010 and 2023 in Germany: a quantitative content analysis
Source: Front Sports Act Living. 2024 Oct 8;6:1446680. doi: 10.3389/fspor.2024.1446680 (PMC11493659; doi:10.3389/fspor.2024.1446680)
Supplement: Supplementary file 1 [file Table1.docx]

*Supplementary Table 1. List of search terms applied.*

| Search terms regarding mental health issues  (*in German*) | psychisch* ODER „mentale Gesundheit” ODER „psychische  Gesundheit” ODER „psychische Erkrankung” ODER „psychische Krankheit*“ ODER „psychische Störung“ ODER „psychische Probleme“ ODER „mentale Probleme“ ODER „mentale Krankheit“ ODER „psychisch krank“ ODER „psychisch erkrankt“ ODER Depress* ODER Burnout* ODER Angststörung* ODER Essstörung* ODER „Anorexia nervosa“  ODER Magersucht ODER Zwangsstörung* ODER „Bipolare Störung*“ ODER „Psychotische Störung“ ODER Psychose* ODER Suizid* ODER Selbsttötung* ODER Selbstmord* ODER Erschöpfung* ODER manisch ODER Panikattacke ODER Missbrauch ODER Bulimie ODER „mentaler Druck“ ODER „mentale Schwäche“ ODER „mental schwach“ ODER „mental instabil“ ODER „Anorexia athletica“ ODER Alkoholsucht ODER alkoholsüchtig ODER drogenabhängig* ODER Drogenmissbrauch ODER Alkoholmissbrauch ODER süchtig ODER drogensüchtig* ODER alkoholabhängig* ODER erschöpft* ODER Manie |
| --- | --- |
| Search terms related to high-performance sports  (*in German*) | Fußball* ODER Bundesliga* ODER Olympisch* ODER  Profisport* ODER Hochleistungssport* ODER Spitzensport* ODER Sportler* ODER Athlet* ODER Spieler* ODER Nachwuchssport* ODER Olympia* |
