# Supplementary material for: Exploring print media coverage of elite athletes’ mental illness between 2010 and 2023 in Germany: a quantitative content analysis
Source: Front Sports Act Living. 2024 Oct 8;6:1446680. doi: 10.3389/fspor.2024.1446680 (PMC11493659; doi:10.3389/fspor.2024.1446680)
Supplement: Supplementary file 2 [file Table2.docx]

*Supplementary Table 2. Explanation of the variables.*

| *Variable* | *Outcome items* |
| --- | --- |
| Year published | 2010 - 2023 |
| News media | F.A.Z.; SZ; Die Zeit; Die Welt; Der Spiegel; Focus; Stern; taz; BILD; SportBILD; BUNTE |
| Article genre | News; feature; interview; comment/column |
| Central thematic focus of the article | Current condition; end of career; portrait of life; abuse; disclosure; suicide; therapy/treatment; injury; unexpected event/behavior; neurological disorder; hospitalization; criticism of the system; description of the system; change of the system |
| Reported mental disorder | Depression; burnout; panic disorder; substance use disorder (alcohol abuse/drug abuse); behavioral addiction; anxiety disorder; bipolar disorder; obsessive-compulsive disorder; attention deficit hyperactivity disorder; posttraumatic stress disorder; eating disorder |
| Citing the person affected by mental illness | Yes/no |
| Citing a mental health expert | Yes/no (Defined as a person with specific education in the field of medicine, sports science or psychology.) |
| Citing the (social) environment | Yes/no (Includes family, friends, teammates and colleagues, as well as people without any contact to the person affected, e.g., politicians, journalists, authors.) |
| Helpline information  (Element of responsible journalism) | Yes/no (e.g., websites, institutions, foundations) |
| Statistics  (Element of responsible journalism) | Yes/no (Data and facts that are scientifically reliable and well-founded, which provide background information for the reader.) |
| Perspective of a mental health expert  (Element of responsible journalism) | Yes/no |
| Adequate language  (Element of responsible journalism) | Yes/no (Avoiding metaphorical terms, as well as colloquial and derogatory expressions. Concerning reports about suicide, providing detailed descriptions about the method or location of committed suicide is identified as inappropriate language.) |
| Criticism of the System | Yes/no (Any critical statement, quote or expression, indicating disapproval or concerns relating to the established framework of the elite sports system.) |
| Change of the System | Yes/no (Describes suggestions, recommendations or ideas aiming to promote awareness, stimulate debate or advocate for positive change concerning mental health.) |
