# Supplementary material for: Exploring print media coverage of elite athletes’ mental illness between 2010 and 2023 in Germany: a quantitative content analysis
Source: Front Sports Act Living. 2024 Oct 8;6:1446680. doi: 10.3389/fspor.2024.1446680 (PMC11493659; doi:10.3389/fspor.2024.1446680)
Supplement: Supplementary file 3 [file Table3.docx]

*Supplementary Table 3. Description of the different central thematic focuses of the articles.*

| *Outcome item* | *Description* |
| --- | --- |
| Portrait of life | Often, childhood and youth are included in this item portraying first experiences in the world of high-performance sports. Environmental conditions such as family and social background are incorporated. The greatest achievements are described as well as low points within life or career. |
| End of career | This item focuses on athletes, who are about to retire or who are facing their final competition before ending their sports career, potentially leaving behind a significant legacy (e.g., Sven Hannawald, Jan Frodeno). Articles often contain retrospective reflection on certain aspects of the career that may have been particularly demanding or led to the retirement/career ending (e.g., burnout, injury). |
| Disclosure | Articles narrating the act of revealing and, consequently, sharing details or facts concerning personal mental health issues that were previously unknown are coded as “disclosure” (e.g., unexpected events like experienced panic attacks during a tournament as well as announcing a break from participation in sports). |
| Criticism of the system | Captures any critical statement or quote, expressing disapproval with embedded values or conditions within the world of high-performance sports. Critique can be related to individual, organizational or societal level (e.g., revealing the strategic cover-up of cases of abuse, suffering from pressure). |
| Change of the system | A “change of the system” is identified as evolution, transformation, adaptation, or modification of the system´s structure or framework. It might be portrayed as a shift, alteration, or reconfiguration in the system´s, practices, or procedures. (e.g., prohibiting head-to-head hits in the NFL, incorporating more sports psychologists in club sports). |
| Description of the system | Articles addressing how the sports world deals with the topic of mental health/illness, what it means to be a professional athlete, the challenges that originate from expectations and the (social) environment within sports, as well as descriptions of the daily routine, training, and the social life of athletes. |
| Abuse | Articles are coded as “abuse” if they focus on the psychological repercussions for the athlete, rather than, for example, systematic criticism as in the case of the scandal concerning the USA gymnastics association. This also includes instances of abuse in the past during childhood or adolescence, perpetrated by family members, the social environment, or coaches, leading to mental health problems still persistent in the present. |
| Injury | An incurred injury or accident has led to mental problems. This encompasses both, long-lasting as well as short-term injuries and their psychological consequences. Examples include a forced break due to injury (and the mental consequences), withdrawal from a significant tournament due to injury (and the mental consequences), and long-standing or recurrent injuries that cause mental distress for the athlete. |
| Suicide | On the one hand, often described as an event that came unexpectedly and shocked the public, presenting uncertainty about the athlete´s well-being before committing suicide and his or her reasons thereof. On the other hand, suicide can also be the result or culmination of a prolonged history of illness (e.g., the suicide of soccer player Andreas Biermann after suffering from depression over years). The article primarily focuses on the suicide itself rather than the system and its conditions in general. |
| Current condition | The interview or report takes place during the ongoing season - for instance, before or after an important competition or tournament. The athlete is interviewed about previous experiences or future events, with a focus on personal or general mental health/attitude (e.g., previous injuries or mental health issues, family situation). |
| Unexpected event or behavior | Any event described in an article that occurred unexpectedly and deviates from socially accepted or conventional behavior/rules (e.g., soccer player Breno is accused of setting his house on fire, Michael Phelps driving drunk, description of an attack on the team bus of Borussia Dortmund and the consequences thereof for the athletes). |
| Neurological disorders | Articles containing neurological disorders in this study are always associated with mental consequences thereof (e.g., head-to-head hits in the NFL, leading to frequent concussions and subsequently physical and mental disorders). |
| Treatment | Is the article primarily focused on therapy? For instance, does it report on how successful or challenging the treatment was for an athlete? If therapy is only mentioned in passing or in the background, the article often falls into the category of “Disclosure”. |
| Hospitalization | Is the article primarily focused on hospitalization? For instance, does it report on how successful or difficult the hospital stay was for the person affected? It can also involve a more socially oriented discussion, such as reporting that hospitalization is still stigmatized. |
